# Deep reinforcement learning-aided autonomous navigation with landmark generators

**DOI:** 10.3389/fnbot.2023.1200214

**Published:** 2023-08-22

**Authors:** Xuanzhi Wang, Yankang Sun, Yuyang Xie, Jiang Bin, Jian Xiao

**Affiliations:** Department of Integrated Circuit Science and Engineering, Nanjing University of Posts and Telecommunications, Nanjing, China

**Keywords:** deep reinforcement learning (DRL), mobile robots, trajectory planning, autonomous navigation, simultaneous localization and mapping

## Abstract

Mobile robots are playing an increasingly significant role in social life and industrial production, such as searching and rescuing robots, autonomous exploration of sweeping robots, and so on. Improving the accuracy of autonomous navigation of mobile robots is a hot issue to be solved. However, traditional navigation methods are unable to realize crash-free navigation in an environment with dynamic obstacles, more and more scholars are gradually using autonomous navigation based on deep reinforcement learning (DRL) to replace overly conservative traditional methods. But on the other hand, DRL's training time is too long, and the lack of long-term memory easily leads the robot to a dead end, which makes its application in the actual scene more difficult. To shorten training time and prevent mobile robots from getting stuck and spinning around, we design a new robot autonomous navigation framework which combines the traditional global planning and the local planning based on DRL. Therefore, the entire navigation process can be transformed into first using traditional navigation algorithms to find the global path, then searching for several high-value landmarks on the global path, and then using the DRL algorithm to move the mobile robot toward the designated landmarks to complete the final navigation, which makes the robot training difficulty greatly reduced. Furthermore, in order to improve the lack of long-term memory in deep reinforcement learning, we design a feature extraction network containing memory modules to preserve the long-term dependence of input features. Through comparing our methods with traditional navigation methods and reinforcement learning based on end-to-end depth navigation methods, it shows that while the number of dynamic obstacles is large and obstacles are rapidly moving, our proposed method is, on average, 20% better than the second ranked method in navigation efficiency (navigation time and navigation paths' length), 34% better than the second ranked method in safety (collision times), 26.6% higher than the second ranked method in success rate, and shows strong robustness.

## 1. Introduction

Mobile robotic devices are becoming increasingly popular in daily life and hazardous industrial production environments (Niloy et al., 2021). In a complex and dynamic environment, the ability of mobile robots to navigate autonomously, reliably, and safely is one of the most concerning issues in academia and industry around the world. In an ordinary static environment, traditional navigation methods such as the Dynamic Window method (DWA) (Seder and Petrovic, 2007), the Timing Elastic Band method (TEB) (Yu et al., 2022), and Model Predictive Control (MPC) (Yu et al., 2020) can perform the task well. But when faced with an environment full of dynamic obstacles, they didn't perform as well (Rösmann and Hoffmann, 2017). Traditional navigation methods are based on artificially designed criteria for avoiding obstacles and safety restrictions. They move straight when there is no obstacle and circle around when encountering impediments (Li et al., 2017), so it is difficult to avoid moving blocks and easily get stuck in stagnation.

Therefore, some researchers have explored a navigation method based on end-to-end deep reinforcement learning (DRL) (Zhu and Zhang, 2021), which directly maps the data obtained by the fuselage sensor to the robot's output actions and can teach the robot complex behavioral rules. Hence, DRL can show high robustness in the face of dynamic interference. Moreover, they demonstrate a high level of generalization in different testing environments. However, the robot autonomous navigation method based on deep reinforcement learning currently has a significant bottleneck (Dugas et al., 2021). In the training process, the network model cannot remember long-term information, so it is difficult to include the initial environmental state information into the training scope at the later stage, which significantly increases the training time. At the same time, due to over-reliance on the generalization ability of the action network, when the generalization ability cannot meet the demand, it is easy to fall into local minima^1^ in the process of long-distance navigation (Zeng et al., 2019).

**Figure 1:**
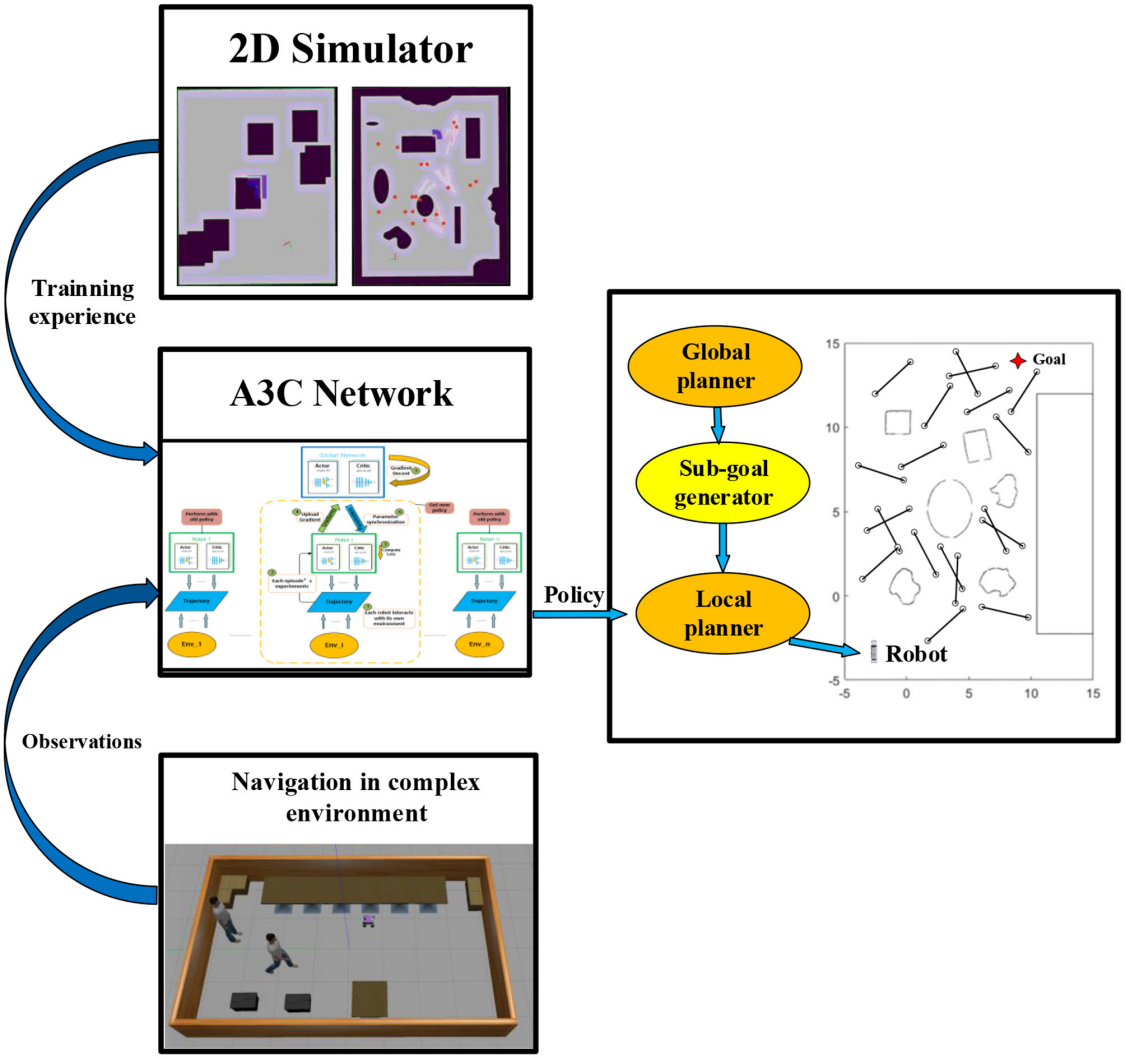
General framework. In the right half, the global planner and the sub-goal generator offer landmarks to the local planner. The left half reveals the DRL-based local planner's operating principle: after getting sufficient training experiences in the 2D simulator, the A3C network get observations from real environments and outputs policies to get the robot to the final goal.

Considering it is an enormous burden for DRL networks to directly map the data obtained by the fuselage sensors to the robot's output actions, we propose a navigation framework, as shown in Figure 1, which splits long-distance navigation into global and local navigation modules connected by a waypoint generator. In our research, this navigation framework is fully integrated into the Robot Operating System (ROS). Based on the Rapidly-exploring Random Trees algorithm (RRT) (Wu et al., 2021) to complete the initial static environment mapping, the specific navigation implementation steps are as follows: First, the A^*^ (Guo et al., 2022) or Dijkstra (Gao et al., 2019) algorithm is deployed to realize the global path planning; Then, the mobile robot finds the most suitable sub-goal on the global path utilizing the real-time sub-goal generation algorithm. Finally, a local planner based on deep reinforcement learning navigates the mobile robot to the sub-goal. After completing several sub-goal navigations, the robot can eventually reach the target point. During local navigation using reinforcement learning, our research set up a suitable rewarding mechanism for mobile robot motion strategy output. The mobile robot successfully arriving at landmarks or maintaining a safe distance with obstacles will receive rewards with a positive value. As for collisions, getting stuck, and being too close to obstacles, those will lead to receiving rewards with a negative value. In pursuing reward maximization, the robot meets the system's navigation and obstacle avoidance needs.

The framework designed in this paper was trained in a ROS 2D simulator. At the same time, under the same test environments, we deployed the existing common traditional navigation algorithm and end-to-end deep reinforcement learning navigation algorithm and compared them with our method. Experimental results demonstrate that our approach exhibits superior navigation efficiency in scenarios with numerous dynamic obstacles and fast-moving barriers compared to the second-ranked method. Furthermore, it outperforms the second-ranked method regarding safety and success rate, displaying significant robustness.

The main contributions emphasize in 3 parts:

a) We propose a new framework for robot navigation. Compared with the DRL-based end-to-end method, which uses original sensor data as input and outputs control strategy, we combine DRL with traditional navigation methods, splitting the tedious long distance navigation task into global navigation based on numerical optimization and local navigation based on DRL. It also reduces the difficulty of training.
b) We design a sub-goal generator connected to two modules. It will generate several high-value landmarks on the global path as targets of local navigation. This method is deployed to enhance the ability of obstacle avoidance under complex dynamic environments.
c) According to the traditional reinforcement learning algorithm lacks long-term memory, our research improves the DRL network's feature extraction module, using the fully connected layer for feature extraction, then linking a memory module, which stores early characteristics for subsequent training. Thus, the generalization ability of the system can be improved to avoid falling into local minima in long-distance navigation.

The structure of this paper is as follows: Section 2 introduces the related work of traditional navigation and agent navigation based on DRL. In Section 3, algorithms designed in this research are described in detail. Then, Section 4 introduces the actual deployment process of our method. Comparing it with different methods, we identify the advantages and disadvantages of the results, and analyzing them in this section. Finally, in Section 5, the work of this paper is summarized, and possible improvements in the future are discussed.

## 2. Related work

### 2.1. Traditional navigation

Autonomous navigation of mobile robots has been extensively studied in many papers. The most common method is to obtain map information through the fusion of sensor data and robot pose information. Then, they perform numerical optimization or geometric optimization based on the map information (Musso and Dambreville, 2021) to find an optimal global path. For example, Bounini et al. (2017), imitating the concepts of electric potential and electric field force in physics, proposed the obstacle avoidance control method of artificial potential field, established the virtual potential field in the robot workspace, set the obstacle as repulsive force and the target as gravity according to the direction of the virtual potential field force, and realized local path planning by finding the gradient of the maximum direction of gravity. However, this method has great disadvantages. When there are obstacles around the target, the attraction of the target will be significantly disturbed by the repulsive force. This makes it difficult for the robot to reach the target point. Comparatively speaking, the Probabilistic Road Map method (PRM) based on graph search (Alarabi and Luo, 2022) can effectively solve the problem of chaotic obstacle distribution. This algorithm establishes probabilistic road maps in the robot's free configuration space (C space) (Lozano-Perez, 1990) by generating and interconnecting a large number of random configurations. These road maps are used to reply to the robot's queries about path planning: Given the initial and final configuration of the robot, PRM connects them to the road map via simple paths (such as straight lines), and then searches for a series of road maps from one connection node to another.

Although the above methods can effectively solve the navigation problem in static environments, they have the same basic idea as other traditional methods, generally establishing the path planning method according to conservative security criteria and strictly implementing the planned global path for navigation. Therefore, in the face of dynamic environments, they often do not perform as well as in static environments (Xu et al., 2022).

### 2.2. Navigation based on DRL

Reinforcement learning (Farhad and Pyun, 2019) simulates the human brain's learning process, allowing agents to constantly interact with the environment and update the neural network according to the feedback obtained from the interaction to output the most appropriate decision to pursue the positive feedback with higher value. Therefore, it is very suitable for solving the autonomous navigation of robots in dynamic changing environments. In recent years, reinforcement learning has achieved excellent results in several fields like robot arm grasping (Inoue et al., 2017) and robot exploration and mapping in unknown areas (Li et al., 2019). In the field of autonomous navigation, the commonly used reinforcement learning method can be summarized as follows: the robot integrates its own information collected by sensors (odometer, LiDAR, camera, etc.) with the environmental information, and directly maps the next action of the robot, so as to complete the navigation to the target point. In the field of autonomous navigation, the commonly used reinforcement learning method can be summarized as follows: the robot integrates its information collected by sensors (odometer, LiDAR, camera, etc.) with the environmental data and directly maps the following action of the robot, to complete the navigation to the target point. Chen et al. (2017a) used DRL to develop a time-saving navigation strategy that complied with standard social norms, such as stopping at the red light and proceeding at the green light. And then, it identified pedestrians on the road through semantic segmentation and collected pedestrian movement information to realize fully autonomous navigation of robot vehicles moving at human walking speed in a downtown environment. Later, for the case of multiple robots, Chen et al. (2017b) also proposed a distributed multi-agent navigation obstacle avoidance algorithm based on deep reinforcement learning, which developed a value network. The collision-free velocity vector is output by the input of the estimated time to reach the target, the joint configuration of the primary and side robots (position and velocity), and the uncertainties in the motion of other agents were also taken into account. Simulation results show that compared with the latest dynamic collision avoidance strategy–Optimal Reciprocal Collision Avoidance (ORCA) (Guo et al., 2021), its path quality (i.e., time to reach the target) is improved by more than 26%. Everett et al. (2018) extended the previous approach. They completed the navigation of multi-agent groups on the road under the premise of mutual obstacle avoidance among various dynamic agents. Without assuming they follow specific behavior rules, they directly mapped the obstacle avoidance strategy by inputting mutual location and environmental information. The algorithm also introduces a strategy using Long-Short Term Memory (LSTM) (Khataei Maragheh et al., 2022), which enables the algorithm to use any number of observations from other agents rather than the fixed numerical size of the primary agent. It is worth noting that such a memory module effectively inherits comprehensive and multi-time data. Therefore, we consider designing a similar memory module to store the input features or the reward value of reinforcement learning to avoid the lack of long-term memory for long-distance navigation.

The above methods are based on end-to-end deep reinforcement learning. The only input information is the sensor's data, which often leads to lengthy training and less reward value. In 2018, Google proposed the combination of the probabilistic road map method and reinforcement learning, hierarchically combining sample-based path planning and reinforcement learning to complete remote navigation tasks, known as PRM-RL (Faust et al., 2018). Firstly, agents are taught to learn short-range point-to-point navigation strategies, which are only constrained by robot dynamics and tasks, and do not need to understand large-scale map topologies. Next, the sample-based planner provides a road map to connect the robot configuration, and by advancing toward these sampling points, it can successfully navigate to the intended goal. This is also the closest approach to our research.

Inspired by the practice of Google, we divide the whole path planning process into global and local planning. We use a common numerical optimization algorithm to generate the global path, and then use deep reinforcement learning as the local planner. This is to reduce the computational burden of the deep reinforcement learning network by offering sub-goals. The most significant difference between our method and Google's work is that Google adopts the K-Neighborhood algorithm (Ran et al., 2021) to sample points with high information value in the static probabilistic road map as landmarks. However, this sampling method cannot meet the requirements in a highly dynamic environment where the spatial region changes rapidly. Therefore, we design a dynamic real-time sub-goal generator. The specific algorithm is described in Section 3.1. In addition, different from previous navigation methods based on DRL, we adopt an Asynchronous Advantage Actor-Critic network (A3C) (Mnih et al., 2016) for reinforcement learning. Actor networks output the robot's motion strategy, while critic networks evaluate whether the motion is appropriate or not at this moment. A3C uses multiple agents, each of which learns in parallel using different exploration strategies in a replica of the actual environment. Finally, a global agent is formed by integrating these experiences to output overall strategies and evaluations. Compared with traditional DRL methods, A3C has better effects and accuracy in continuous and discrete behavior space (Liu et al., 2020). The algorithmic details are described in Section 3.3.

## 3. Methodology

### 3.1. Problem definition

When our mobile robot faces with an unfamiliar environment, the first thing to do is to build a map for it. With the rapid development of Simultaneous Localization and Mapping (SLAM), efficient positioning and mapping have become a reality. Before setting up dynamic obstacles, we utilize RRT algorithm to quickly complete the construction of static environment's map. Then, the A^*^ algorithm acts as a global planner to plan the first global trajectory.

**Figure 2:**
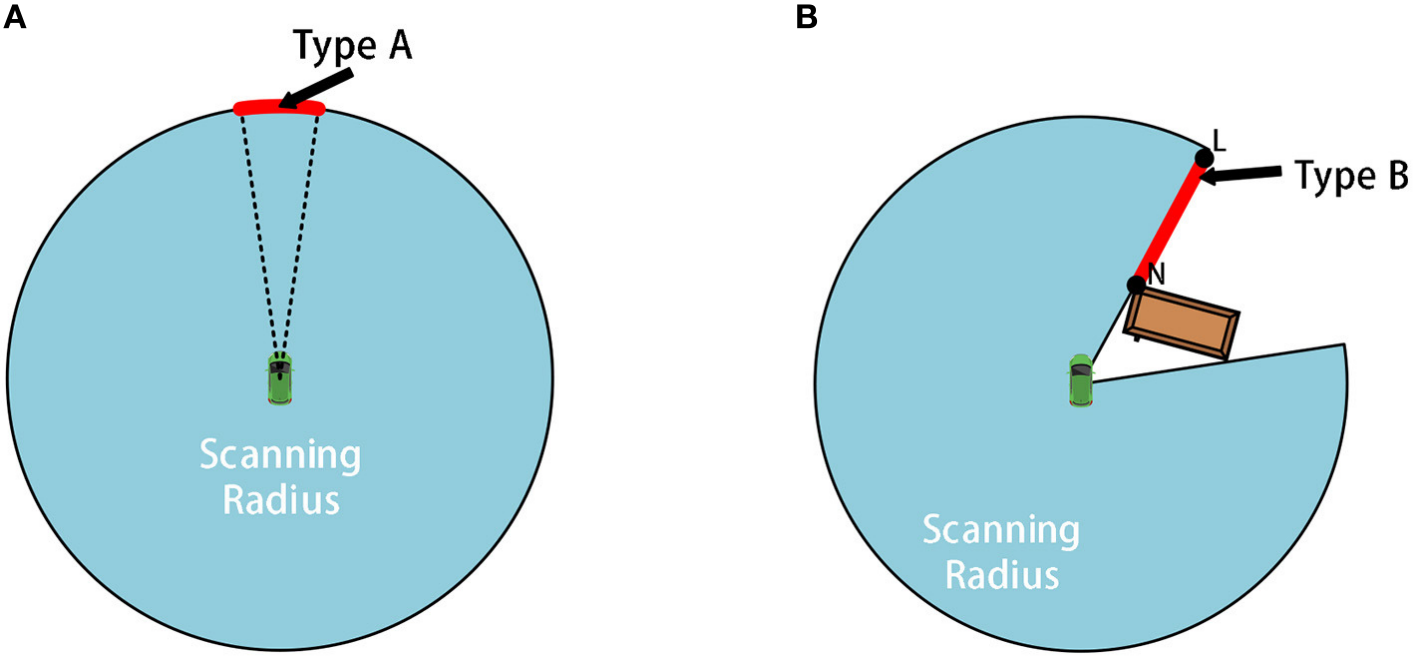
The schematic diagram for frontiers of type A and B: **(A)** shows schematic diagram of A-type frontier while **(B)** shows schematic diagram of B-type frontier.

Our innovation is in the next designs. As shown in Figure 2, we add dynamic obstacles to the environment. When the mobile robot starts approaching the destination based on the first global path, what the landmark generator needs to do is to find the most appropriate sub-goal considering various obstacles. At the same time, the reinforcement learning algorithm will act as a local planner to output policies for mobile robots. The details of the algorithm are as follows:

### 3.2. Real-time sub-goal generator

Unlike Google's K-neighborhood sampling, the sub-goal generated by the landmarks generator in our research depends on the current position of the mobile robot, which effectively improves the limitation that K-neighborhood sampling can only be effective in a fixed environment. The following sections provide an explanation of the sub-goal generator algorithm.

#### 3.2.1. Preprocessing raw LiDAR's data

When ROS Gazebo reads data from the Lidar topic, it often reads invalid “inf” or “nan” values. In this case, “inf” indicates that the distance value is infinite and “nan” indicates that no data is available.

Let *d*[*i*] represent the range value No. i in the original laser data, *d*_max_ and *d*_min_ represent the farthest and nearest effective range values of LiDAR, respectively, and *size* indicates the number of original laser data. The preprocessing method is as follows:

a) Traverse each raw laser data *d*[*i*] sequentially, and if *d*[*i*] is less than *d*_min_, *d*_max_ is assigned to *d*[*i*]. The index number of the first valid data is denoted as *j*;
b) Make *i* equal to *j*, if the 0th data *d*[0] in the original laser data is invalid, assign *d*[0] as *d*_max_; If the last data *d*[*size*−1] in the original data is invalid, assign *d*[*size*−1] as *d*_max_;
c) Make *i* equal to *i*+1. If *d*[*i*] is invalid data and *i* is less than *size*−1, skip to step (d); If *d*[*i*] is valid data and *i* is less than *size*−1, repeat step (c); If *i* is equal to or greater than *size*−1, make *i* equivalent to *j* and skip to step (e);
d) If both adjacent data to *d*[*i*] are valid data, assign the smaller value of the two data to *d*[*i*]; If there is only one valid data adjacent to *d*[*i*], assign that valid data to *d*[*i*]; If *i* is greater than *j*, skip to step (c); otherwise, skip to step (e);
e) Make *i* equal to *i*−1. If *d*[*i*] is invalid data and *i* is greater than 0, skip to step (d); If *d*[*i*] is valid data and *i* is greater than 0, repeat step (e); If *i* is equal to or less than 0, the preprocessing ends.

#### 3.2.2. Obtaining frontiers

Before getting the sub-goal, we will first detect the frontier of the local environment to narrow the search range. Our method divides the local environmental frontier into two types: A and B.

The A-type frontier is generated by the laser range constraint, located at the maximum range of the LiDAR, as shown by the arc-shaped red line in Figure 2A. The B-type frontier is generated by obstacles, as shown by the red line NL marked in Figure 2B. The length of both types of frontiers is longer than the width of the mobile robot to ensure that the mobile robot can cross the frontier.

First, assume that the frontier set in the current local environment is *F*_*c*_,


(1) $$\begin{array}{l} F_{c}=<F_{0},\ldots ,F_{i}> \end{array}$$

For each frontier, there are:


(2) $$\begin{array}{l} F_{i}=<idx_{s},idx_{e},type> \end{array}$$

In this formula, *idx*_*s*_ is the index number corresponding to the starting point of the frontier in the laser data, *idx*_*e*_ represents the index number corresponding to the ending point of the frontier in the laser data, and *type* represents the type of the frontier. Define the length of type A frontier as:


(3) $$\begin{array}{l} length_{A}=f_{\max}\cdot angle\text{\_}inc\cdot \left(idx_{e}-idx_{s}\right) \end{array}$$

*f*_max_ which sets by ourselves represents a number slightly smaller than *d*_max_, *angle*_*inc* is the LiDAR's angular resolution.

We first sequentially traverse the preprocessed laser data. For each laser data segment with a distance value greater than *f*_max_, we use *idx*_*s*_ and *idx*_*e*_ to determine the index numbers of the beginning and end of the data segment, and then calculate its length according to formula (3). If the length is greater than the width of the mobile robot, a type A frontier is detected and < *idx*_*s*_, *idx*_*e*_, *A*>is added to the *F*_*c*_ set. Continue traversing the preprocessed laser data until all laser data has been traversed.

For type B frontier, we redefine the length:


(4) $$\begin{array}{l} length_{B}=|d\left[idx_{e}\right]-d\left[idx_{s}\right]| \end{array}$$

For any two adjacent laser data, record their index numbers using *idx*_*e*_ and *idx*_*s*_ respectively, and calculate the *length*_*B*_. If the length is greater than the width of the mobile robot, a type B frontier is detected and < *idx*_*s*_, *idx*_*e*_, *B*> is added to the *F*_*c*_ set. Continue traversing the preprocessed laser data until all laser data has been traversed.

#### 3.2.3. Obtaining sub-goals from frontiers

Considering the different reasons for frontier generation in types A and B, our algorithm utilizes different geometric rule sets to obtain corresponding sub-goals.

Assuming the current moment is *t*, let the set of sub-goals at the current moment be:


(5) $$\begin{array}{l} P_{c}^{t}=<p_{0},\ldots ,p_{i}> \end{array}$$

For each sub-goal *p*_*i*_:


(6) $$\begin{array}{l} p_{i}=<position,d,idx,type_{p}> \end{array}$$

In this formula, *position* = < *x, y*> is the global coordinate of the sub-goal, *d* represents the distance between *p*_*i*_ and the center of the LiDAR, *idx* represents the index number of the direction of the sub-goal in the laser data, *type*_*p*_ is the type of the current sub-goal.

**Figure 3:**
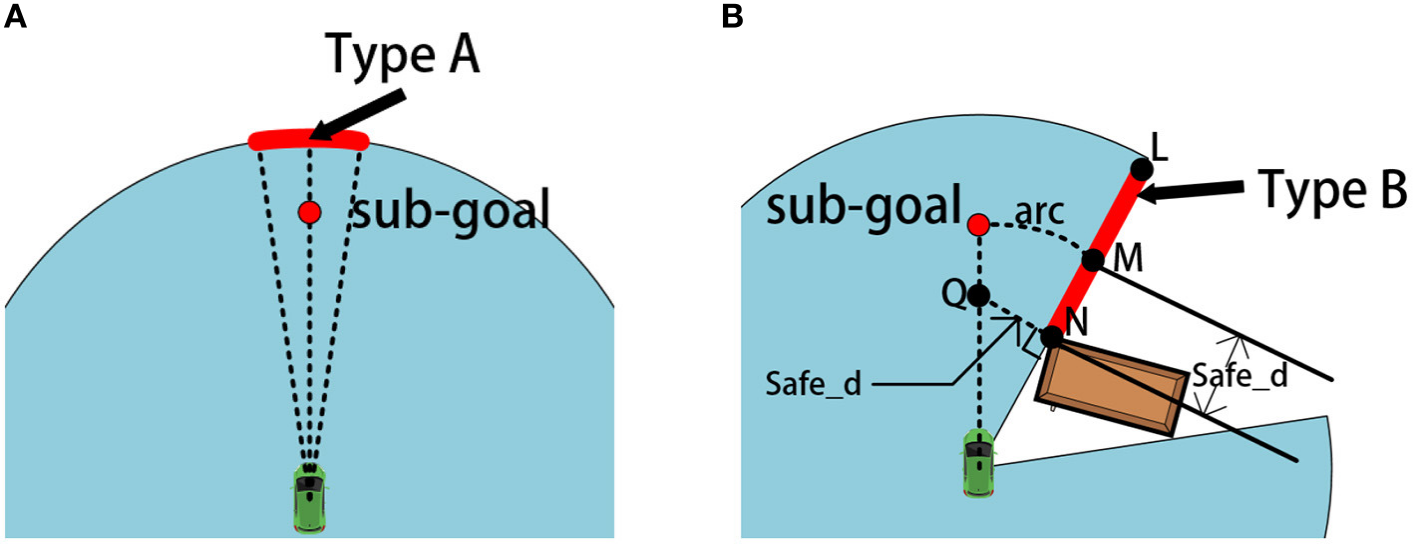
The schematic diagram for selecting sub-goals of type A and B: **(A)** shows schematic diagram of a A-type sub-goal while **(B)** shows schematic diagram of a B-type sub-goal.

The obtained rules for type A sub-goals are as follows:

a) Connect the midpoint of the type A frontier to the center of the LiDAR;
b) Take *p*_*i*_ on the connecting line as the current initial sub-goal, so that the distance between *p*_*i*_ and LiDAR is *f*_max_, as shown in Figure 3A. Then the various elements of *p*_*i*_ are:

(7) $$\{\begin{array}{c} type_{p}=A\\ idx=(idx_{s}+idx_{e})/2\\ d=f_{\max} \end{array}$$

At this time, the initial sub-goal *p*_*i*_ will be added to the set $P_{c}^{t}$.

We adopt different methods for obtaining the type B sub-goal. First of all, we set a safe distance *safe*_*d* slightly greater than half the width of the mobile robot. The obtained rules for type B sub-goals are as follows:

a) Mark the two ends of type B frontier as N and L respectively;
b) Take a point M on NL, so that MN = *safe*_*d*;
c) Use the center position of the LiDAR as the center of the circle, create an arc *arc* passing through point M;
d) Take a point Q on the perpendicular line passing through N points, so that NQ = *safe*_*d*. And point Q is within the free passage area.
e) Make a ray pointing from the center of the LiDAR toward point Q, and the intersection of the ray and *arc* is the initial sub-goal *p*_*i*_, as shown in Figure 3B.

We set a variable *skip* to control the index of type B sub-goals:


(8) $$\begin{array}{l} skip=\;safe\text{\_}d/\left(angle\text{\_}inc\cdot \left(d-safe\text{\_}d\right)\right) \end{array}$$

At this time, the various elements of *p*_*i*_ are:


(9) $$\{\begin{array}{c} type_{p}=B\\ d=\min\left(d\left[idx_{e}\right],d\left[idx_{s}\right]\right)+safe\_d\\ idx=idx_{e}-skip,if\;d\left[idx_{s}\right]>d\left[idx_{e}\right]\\ idx=idx_{s}+skip,if\;d\left[idx_{s}\right]<d\left[idx_{e}\right] \end{array}$$

And now, the initial sub-goal *p*_*i*_ will be added to the set $P_{c}^{t}$.

So far, two types of sub-goals have been achieved. DRL-based local planner will complete navigation from the initial position to the sub-goal. Once the sub-goal point is reached, the A^*^ algorithm will update the global path to provide the initial orientation for the mobile robot.

### 3.3. Local navigation based on DRL

The autonomous navigation of agents to target goals can be considered as a partially observable Markov decision processes^2^ (POMDP) problem (Liu et al., 2021) which is formally formalized as a 5-tuple (*T, S, O, A, R*, γ): including the current time *T*, the environment state *S*, the information observed by the mobile robot *O*, the motion space of the mobile robot *A*, the reward *R* and the discount rate of the reward γ as time progresses. Reinforcement learning algorithms achieve their goals by maximizing rewards. Considering that the overall modeling of the environment is very heavy, we propose a model-free actor-critic structure to optimize the reinforcement learning algorithm further.

In order to intuitively demonstrate the navigation and obstacle avoidance capabilities of mobile robots, the following state-value function is constructed as follows:


(10) $$\begin{array}{l} V_{\pi }\left(s\right)=\underset{a}{\sum }\pi \left(a|s\right)\cdot Q_{\pi }\left(s,a\right) \end{array}$$

π (*a*|*s*) is a policy function that will output the probability value of the robot's following action. The robot will move according to the maximum probability of the action; *Q*_π_ (*s, a*) is a value function that will output the predicted reward value, which can be used to evaluate whether the robot's action at the current time is conducive to completing the final navigation task. The algorithm uses a neural network π (*a*|*s*; θ) to approximate the policy function π (*a*|*s*); Another neural network *q* (*s, a*; **w**) to approximate the value function *Q*_π_ (*s, a*), θ and **w** are trainable parameters of the corresponding neural network. Thus, the state-value function can be rewritten as:


(11) $$\begin{array}{l} V_{\pi }\left(s;\theta ;w\right)=\underset{a}{\sum }\pi \left(a|s;\theta \right)\cdot q\left(s,a;w\right) \end{array}$$

**Figure 4:**
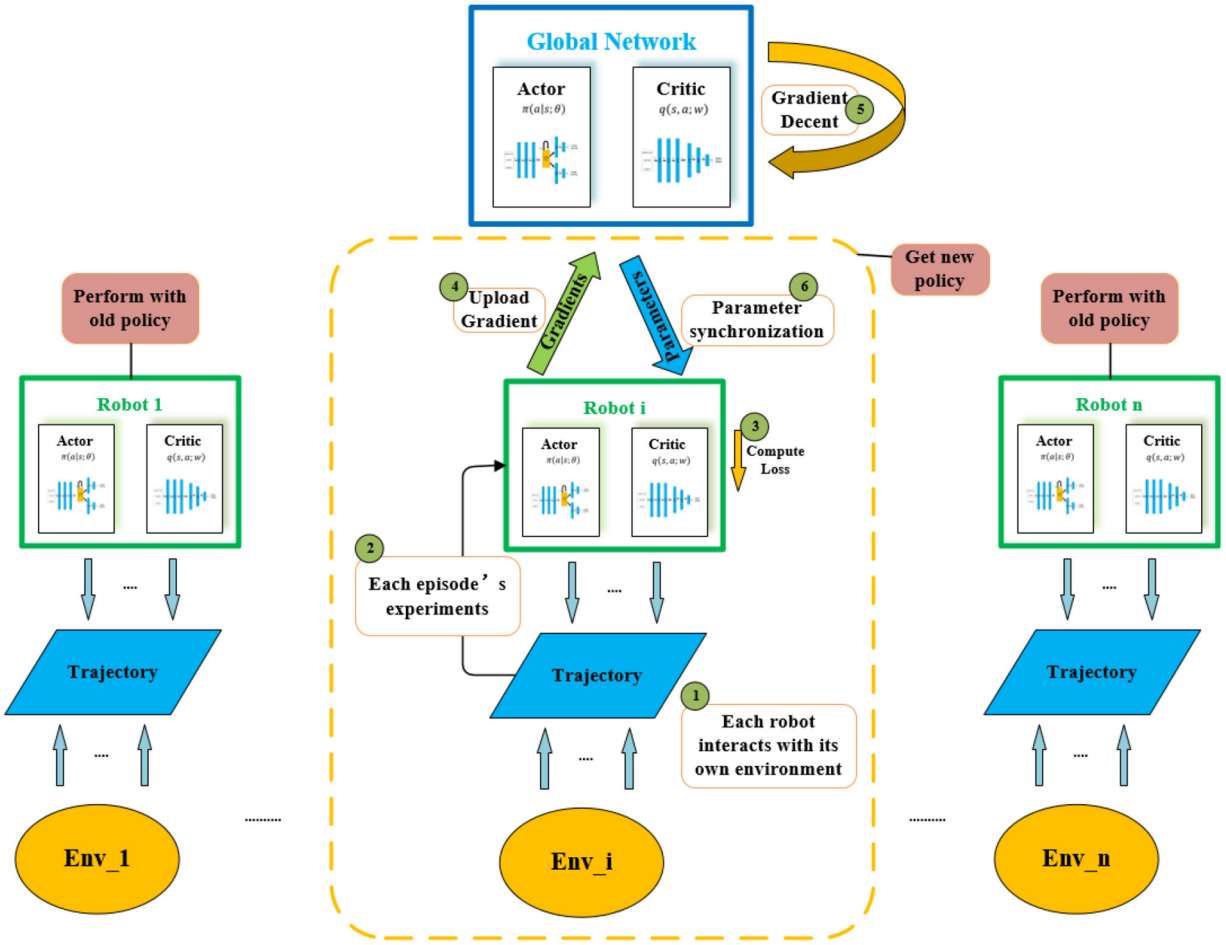
The whole A3C network. Agents are trained asynchronously in each different environment. On each step, the agent gets experiments to the Actor-Critic network after interacting with its own environment. Each agent then transmits the newly acquired policy separately to the global agent.

Take it as an actor-critic reinforcement learning model in one environment, and its value directly reflects the robot's navigation and obstacle avoidance abilities. Several fully connected layers are constructed at the beginning of both networks to enable the neural network to better retain features from the input information for a long time. In addition, a memory module is added behind the policy network. At the same time, to reduce the difficulty of network convergence, we utilize several different environments for training, then input the training experience into a master agent, and construct the asynchronous advantage actor-critic reinforcement learning model (A3C). The integrated reinforcement learning and training process in all asynchronous environments is shown in Figure 4.

#### 3.3.1. Neural network architecture

**Figure 5:**
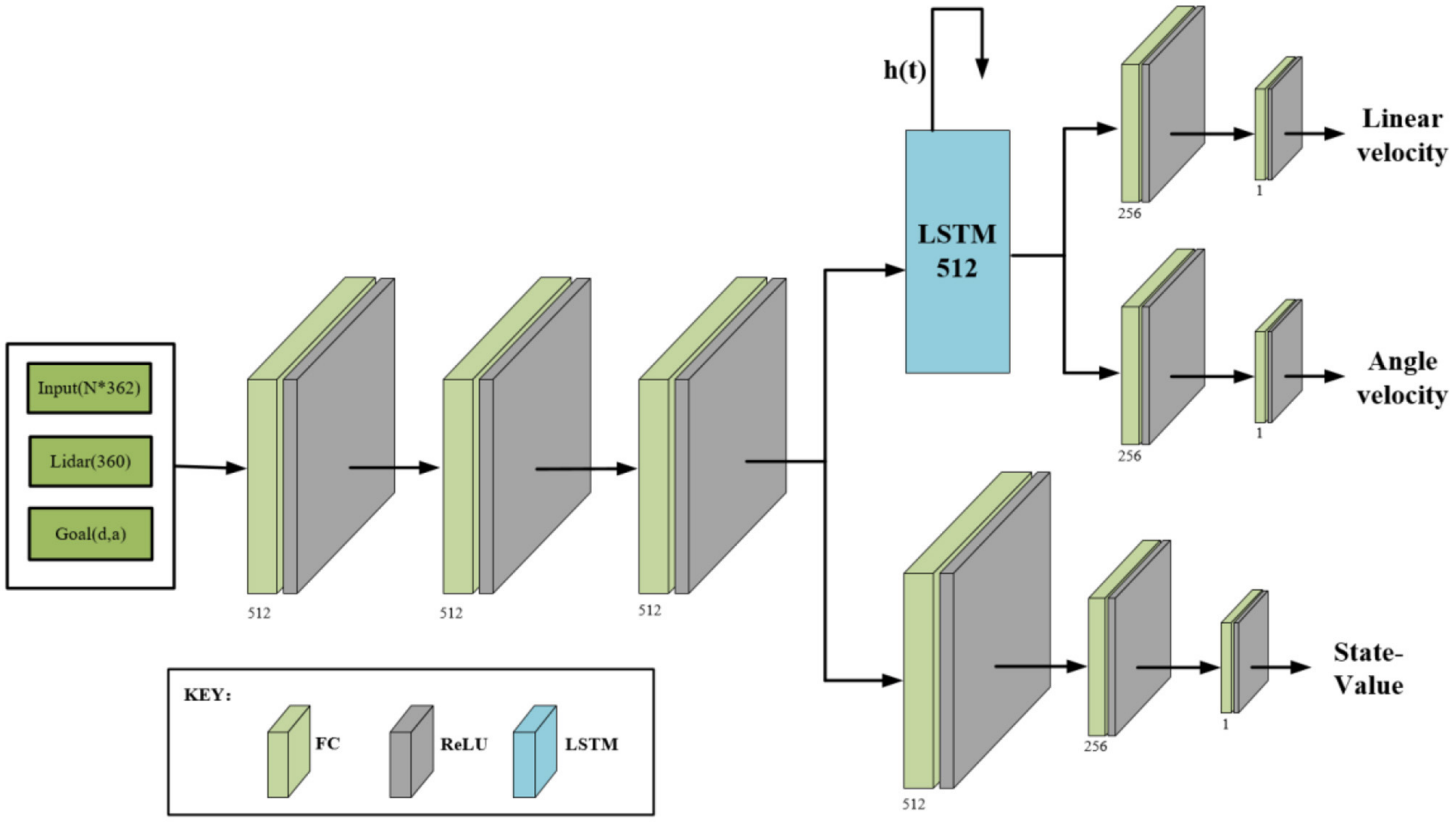
Actor-Critic network model. Input sensor and sub-goal information, output motion policies and the state-value. For both, actor and critic network, we use the same head. The LSTM stores long-term memory for the actor network to make decisions about the long-distance navigation, as depicted in this figure, **h(t)** carries characteristic information repeatedly input to the computing unit.

In any of the environments in Figure 4, the network structure of the actor-critic model is shown in Figure 5. The structure is divided into two parts. The first half is the policy network (actor), used to output policies that the mobile robot takes; The bottom half is the value network (critic), which outputs state-value functions to evaluate how effective or bad the policies are. The two parts use the same backbone to extract the input information's features, including the 360° LiDAR scanning information and the location information of the sub-goal. It should be noted that the sub-goal position information is relative position information, which contains two elements, namely the distance and angle of the sub-goal relative to the mobile robot. The two-part shared backbone feature extraction network adopts three layers, each with 512 fully connected units, and a Rectified Linear Unit (ReLU) (Glorot et al., 2011) is used as the activation function. To avoid long-term memory loss caused by the long-distance transmission of feature information in the network, after the feature extraction module of the critic network is completed, we add a Long-Short Term Memory module (LSTM) containing 512 units (Ordóñez and Roggen, 2016), which is used to store the feature information and accumulated reward values. This is followed by 256 fully connected layers with ReLU activation units, and finally, it ends with a layer with a single neuron that has linear activation to output different actions. In the lower part of the value network, the feature extraction module is followed by 256 fully connected layers with ReLU activation units. It also ends with a single neuron, which is used to output the state-value function.

#### 3.3.2. Networks' iteration

On the one hand, to improve the state-value function *V*_π_ (*s*; θ *w*) reflecting the overall navigation and obstacle avoidance abilities of the system, we need to update π (*a*|*s*; θ) to output better policies; On the one hand, supervision of the quality of the policies can only be realized through the value network's output–the value function *q* (*s, a*; *w*). Therefore, it is necessary to update the value network to estimate the reward of the action policies more accurately. The value network (critic) will only randomly rate the reward at first, but as the training progresses, the value network's judgments become more accurate due to the monitoring of environmental feedback. A complete dual-network iteration process can be summarized as follows: 1. Obtain the current environment state *S*_*t*_; 2. According to the current policy network π (·|*S*_*t*_; θ_*t*_), randomly outputs the action *a*_*t*_; 3. Perform action *a*_*t*_ and observe new state *S*_*t*+1_; 4. In the value network, the Temporal Difference (TD) algorithm (Tesauro, 1995) is used to update the network parameter **w**. In contrast, in the policy network, the policy gradient algorithm is used to update the network parameter θ.

The TD algorithm is a method used to estimate the value function of a policy that can be learned from samples without prior knowledge of the environment. As a first step, the policy network obtains two consecutive actions, *a*_*t*_ and *a*_*t*+1_, through random sampling. Then the value network will evaluate the value functions *q* (*s*_*t*_, *a*_*t*_; *w*_*t*_) and *q* (*s*_*t*+1_, *a*_*t*+1_; *w*_*t*_) corresponding to these two actions. In this way we can get a TD target function:


(12) $$\begin{array}{l} y_{t}=R_{t}+\gamma \cdot q\left(s_{t+1},a_{t+1};w_{t}\right) \end{array}$$

It's the reward at the moment plus the discount rate γ mentioned earlier times the value of the reward estimated by the value network at the next moment. The TD target function *y*_*t*_ is similar to *q* (*s*_*t*_, *a*_*t*_; *w*_*t*_) in that they are both estimates of the sum of future rewards, but *y*_*t*_ is more accurate and reliable because part of it is the actual observed reward *R*_*t*_. The purpose of the TD algorithm is to make the value network predict *q* (*s*_*t*_, *a*_*t*_; *w*_*t*_) is closer to the TD target function. Therefore, we adopt a mean square error to construct the loss function:


(13) $$\begin{array}{l} L\left(\text{w}\right)=\frac{1}{2}{\left[q\left(s_{t},a_{t};w\right)-y_{t}\right]}^{2} \end{array}$$

In order to make the gap between *q* (*s*_*t*_, *a*_*t*_; *w*) and *y*_*t*_ smaller and smaller, gradient descent can be used to iterate the value network parameter **w**:


(14) $$w_{t+1}=w_{t}-\alpha \cdot \frac{\delta L\left(w\right)}{\delta w}{|}_{w=w_{t}}$$

The parameter α is the learning rate of the value network.

To update the policy network parameter θ of the current state so that the state-value *V*_π_ (*s*; θ *w*) that evaluates the quality of the policies becomes as large as possible, the gradient of the state-value function in the θ direction at this moment can be updated by gradient ascent:


(15) $$\begin{array}{l} \theta_{t+1}=\theta_{t}+\beta \cdot \frac{\delta V\left(s;\theta ,{\text{w}}_{\text{t}}\right)}{\delta \theta } \end{array}$$

The parameter β is the learning rate of the policy network. At this time, **w**_**t**_ is a constant value, and the gradient of the state-value function in the θ direction can be expanded as:


(16) $$\begin{array}{l} \frac{\delta V\left(s;\theta \right)}{\delta \theta }=\underset{a}{\sum }\frac{\partial \pi \left(a|s;\theta \right)}{\delta \theta }\cdot Q_{\pi }\left(s,a\right) \end{array}$$

The first half on the right side of the equation is the gradient of the policy function in the θ direction under all action spaces. The second half is the assessment provided by the value function at the moment. However, in practical applications, it is inefficient to carry out gradient updates for the policy function directly because the output range of the policy function is (0, 1). To increase the data range, speed up the policy update speed, and eliminate the correlation in the sample calculation of the same action space (Silver et al., 2016), the identity variation of the equation is as follows:


(17) $$\begin{array}{l} \frac{\delta V\left(s;\theta \right)}{\delta \theta }=\underset{a}{\sum }\pi \left(a|s;\theta \right)\frac{\partial \log_{\pi }\left(a|s;\theta \right)}{\delta \theta }\cdot Q_{\pi }\left(s,a\right) \end{array}$$

Because of the existence of a logarithmic function, the output range of the policy function can become (−∞, 0). Therefore, we define a new policy function:


(18) $$\begin{array}{l} g\left(a,\theta \right)=\frac{\delta log_{\pi }\left(a|s;\theta \right)}{\delta \theta }\cdot Q_{\pi }\left(s,a\right) \end{array}$$

Combined with formula (8), we can find that the gradient of the state-value function in the θ direction is the expectation of this new policy function:


(19) $$\begin{array}{l} \frac{\delta V\left(s;\theta ,{\text{w}}_{\text{t}}\right)}{\delta \theta }=E_{A}\left[g\left(A;\theta \right)\right] \end{array}$$

According to the definition of expectation, *g*(*a*, θ) can be considered as an unbiased estimation of $\frac{\delta V\left(s;\theta \right)}{\delta \theta }$, so *g*(*a*, θ) can be used to update the policy network:


(20) $$\begin{array}{l} \theta_{t+1}=\theta_{t}+\beta \cdot g\left(a;\theta_{t}\right) \end{array}$$

We use two identical Adam optimizers to iterate the parameters of the two networks. The learning rate is 0.0001, and the epsilon is set to 0.003. The discount rate γ for the TD target is set at 0.9.

This is followed by training in continuous motion state for greater flexibility and fluid movement. According to the actual motion mode of Turtlebot3 (Zhao et al., 2020) used in this research, the action space A is formalized as follows:


(21) $$\begin{array}{l} a=\left\{v_{lin},v_{ang}\;\right\},\\ v_{lin}\in \left[0,0.26\right]m/s,v_{ang}\in \left[-2.7,2.7\right]rad/s \end{array}$$

#### 3.3.3. Reward mechanism

To make the training process more efficient, appropriate rewards need to be set for the entire local navigation process. In the reward system, there are positive rewards for successfully reaching the sub-goals and negative rewards for colliding and staying within a dangerous distance of the obstacle. For clarity, we'll denote them as *r*_*s*_, *r*_*d*_, *r*_*c*_, *r*_*st*_. The total reward *R*_*t*_ is calculated as follows:


(22) $$\begin{array}{l} R_{t}=r_{s}^{t}+r_{d}^{t}+r_{c}^{t}+r_{st}^{t} \end{array}$$

(23) $$r_{s}^{t}=\{\begin{array}{c} 50,\;arrive\;at\;subgoal\\ 0,\;otherwise \end{array}$$

(24) $$r_{d}^{t}=\{\begin{array}{c} -0.5,\;\Delta d<\;d_{safe}\\ 0,\;otherwise \end{array}$$

(25) $$r_{c}^{t}=\{\begin{array}{c} -20,\;collisions\;happen\\ 0,\;otherwise \end{array}$$

(26) $$r_{st}^{t}=\{\begin{array}{c} -0.05,\;\Delta r<r_{\min}\\ 0,\;otherwise \end{array}$$

Among these reward conditions, Δ*d* refers to the distance between the mobile robot and the obstacle. In this research, the safe distance *d*_*safe*_ is set as 0.1 m. Δ*r* refers to the relative displacement within a given time of 1 s, and *r*_min_ refers to the shortest moving distance that the system can tolerate for the mobile robot within 1 s, which is set as 0.03 m according to the experience in the experiment.

#### 3.3.4. Training setup

The training process of the agent takes place in randomized environments, as illustrated in Figure 1. Following each episode, walls and static obstacles are randomly generated. Additionally, dynamic obstacles are spawned at random positions and move in unpredictable trajectories. This deliberate randomness serves the purpose of mitigating over-fitting concerns and fostering improved generalization of the agent's learned behaviors.

To accommodate the inclusion of more intricate obstacle models and shapes, application interfaces are provided. These interfaces enable the integration of complex obstacle representations into the training framework, allowing for greater diversity in the training scenarios.

The training curriculum is designed to adapt dynamically based on the agent's performance. When the agent surpasses a predefined success threshold, indicated by the cumulative mean reward, the curriculum increases the number of obstacles spawned in subsequent episodes. Conversely, if the agent's success rate is below the threshold, the curriculum reduces the number of obstacles in order to facilitate a smoother learning process.

## 4. Results and assessments

### 4.1. Deployment of training and environments

As shown in Figure 1, map boundaries and static obstacles are constant, while dynamic obstacles are randomly generated. Based on the characteristics of asynchronous training, we create different copies of the environment for multiple agents to train simultaneously, and these copies become more and more difficult. To alleviate the overfitting problem and enhance the generalization ability of the policy and value network. All training sessions are conducted in the ROS 2D simulator, while the Turtlebot3 vehicle is used to evaluate the navigation strategy of the design entirely.

After training begins, update the difficulty of the training environment based on the success threshold. After every ten successful navigations in the same environment, the environment will increase the difficulty by increasing the number or speed of dynamic obstacles. All the training is done on the PC, which is configured with an Intel i7-9750H CPU. And optimize the training process with a GeForce GTX 1650. Appendix Table 1 lists the training hyperparameters.

### 4.2. Deployment of other navigation methods

To demonstrate the advantages of the proposed navigation framework, a variety of different navigation methods are deployed in the same environment for comparison, including the Dynamic Window Approach (DWA) and the Time Elastic Band method (TEB) based on numerical optimization, as well as a recent obstacle avoidance approach based on DRL: CADRL (Everett et al., 2018).

In addition, to prove the effectiveness of the memory module, we add an additional experimental comparison part to the paper. Compare the navigation framework using the memory module with the framework not using the module.

### 4.3. Carry out experiments

**Figure 6:**
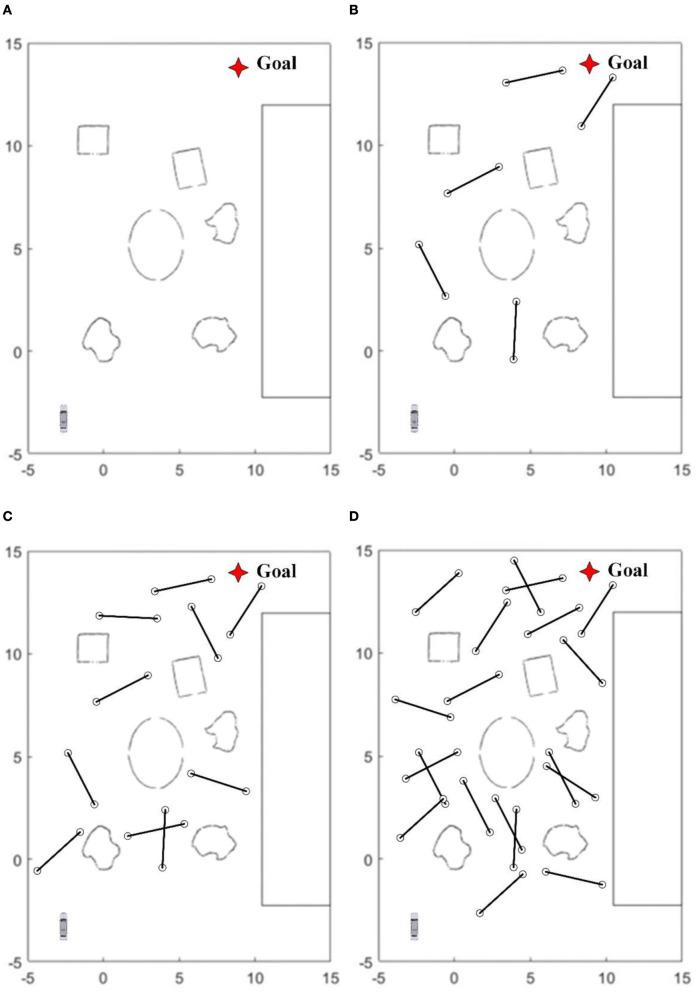
In test environments, **(A)** has only static obstacles. From **(B–D)** 5, 10, and 20 dynamic obstacles moving back and forth at a certain speed between two points 3 meters apart are configured, respectively.

To test the navigation and obstacle avoidance abilities of mobile robots in different environments, four test environments of various levels are constructed in this paper, as shown in Figure 6.

**Figure 7:**
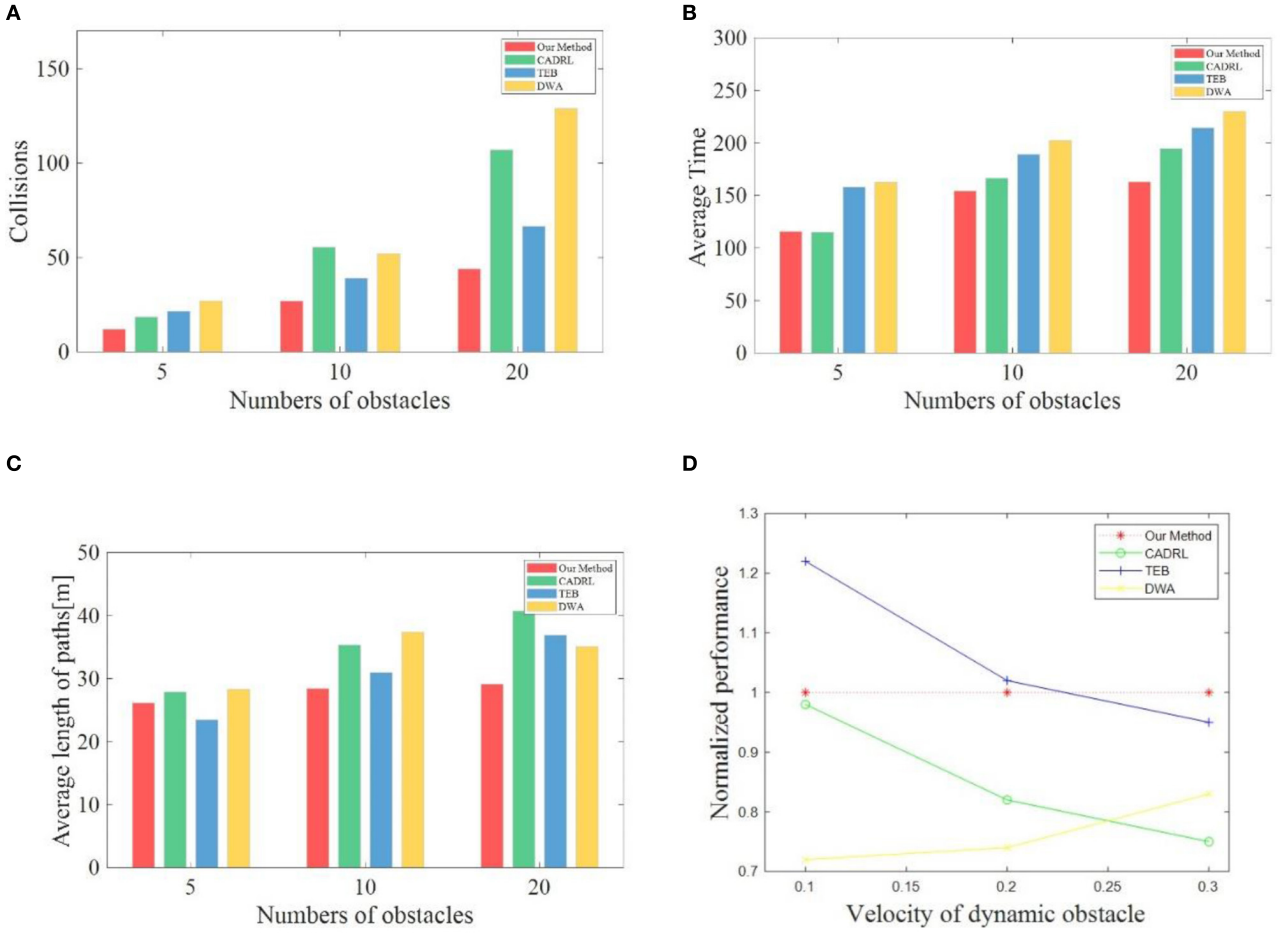
**(A–C)** Shows the performance of each method at a dynamic obstacle velocity of 0.3 m/s. **(D)** Shows the relative performance of all the methods.

In this research, different algorithms are deployed on the robot and tested. We set the running quantity and speed of different dynamic obstacles during the test and completed 100 tests under each condition. Table 1 records the average time spent, the average path length, the average number of collisions, and overall success rate to compare the various methods' performance comprehensively. It is critical to note that success is determined by both the number of collisions and the elapsed times of the robot. If the running time is more than 4 min or the collisions are more than 4 times, it is judged as a failure. Since there is no robot collision in the static map and all robots have reached the final goal, the navigation performance of each algorithm in the static map is not listed in Table 1 in our research. To display the obstacle avoidance ability of the mobile robot in a high-speed dynamic environment more intuitively, we select the performance of various algorithms when the dynamic obstacle speed *v*_*obs*_ is 0.3 m/s and make bar charts in Figure 7.

**Table 1:** Quantitative evaluations.

	**Time[s]**	**Path[m]**	**Collisions**	**Success**	**Time[s]**	**Path[m]**	**Collisions**	**Success**	**Time[s]**	**Path[m]**	**Collisions**	**Success**
	**5 dyn. obstacles**				**10 dyn. obstacles**				**20 dyn. obstacles**			
*v*_*obs*_ = 0.1*m*/*s*												
Our method	**121.19**	24.09	1	100	**131.69**	23.44	7.5	100	141.85	25.42	14	97
CADRL	132.43	29.22	**0**	100	133.56	28.74	**1**	100	**139.62**	24.17	11.5	94.5
TEB	154.87	**21.67**	**0**	100	169.90	**20.18**	3.5	100	167.23	**23.27**	**6.5**	**98**
DWA	147.03	28,34	9	92	175.31	26.82	31	88	175.98	26.15	72	82
*v*_*obs*_ = 0.2*m*/*s*												
Our method	119.22	23.11	**3.5**	98	**142.17**	24.31	**14**	**95**	**142.57**	26.52	**26**	93
CADRL	**114.57**	24.59	8	**100**	146.98	25.45	37	92	151.84	28.96	42	86.5
TEB	139.94	**22.57**	5.5	96	166.07	**21.20**	22	94	163.48	25.79	36.5	92.5
DWA	152.31	26.41	21	84	174.21	30.71	48	82	167.94	**25.13**	108	54
*v*_*obs*_ = 0.3*m*/*s*												
Our method	115.72	26.13	**12**	**94**	**154.35**	**28.41**	**27**	**92**	**162.84**	**29.13**	**44**	**78.5**
CADRL	**114.91**	27.88	18.5	82	166.38	35.33	55.5	78	194.56	40.74	107	62
TEB	157.91	**23.45**	21.5	91	189.09	30.96	39	86	214.31	36.89	66.5	68
DWA	162.43	28.32	27	77	202.58	37.38	52	76.5	229.95	35.08	129	56

The meaning of the bold values is the numerical value that performs best in the same dynamic environment.

#### 4.3.1. Assessment of navigation capability

To evaluate the navigation capabilities of various algorithms, we mainly compare the average time required to reach the goal each time, the length of the path, and the success rate per hundred tests. For the sake of simplicity, we define the time consumed to reach the target each time as navigation speed and the navigation path length as navigation efficiency. As can be seen in Table 1, TEB shows strong navigation efficiency when there are only five dynamic obstacles. TEB planned the shortest path regardless of the speed of the dynamic obstacle, and the success rate is more than 90%. But at the moment, the TEB algorithm does not show high navigation speeds. TEB is about 30% slower than the two DRL-based methods. When the *v*_*obs*_ is 0.1 m/s, the navigation speed of TEB is even slower than that of DWA. When the *v*_*obs*_ is 0.2 m/s and 0.3 m/s, respectively, the navigation time of the TEB algorithm is the same as that of the DWA algorithm. We speculate that since the TEB algorithm is more dependent on globally planned paths, even if dynamic obstacles occur, following the global approach is still the preferred solution of the algorithm, resulting in increased planning time. At the same time, due to the constraint of the distance information provided by the LiDAR, to avoid collision with obstacles, the robot deploying the TEB algorithm will appear stagnant and shaky, so much time is wasted. However, the two methods based on DRL will try to cross the lines of moving obstacles when they encounter obstacles, so stagnation rarely occurs. In addition, although CADRL shows some advantages in navigation speed when the *v*_*obs*_ is 0.1 m/s and 0.2 m/s, the navigation efficiency and success rate of CADRL are not as good as that of TEB and our method. Our method shows excellent advantages when the *v*_*obs*_ is 0.3 m/s. Although it is exceeded by CADRL in navigation speed and TEB in navigation efficiency and success rate when there are only 5 dynamic obstacles, the success rate of the two methods is lower than that of the method in our research. The success rate of CADRL is even 10% lower than our method's. Especially after the number of obstacles increases, all performances of our method are the best. Compared with conditions with lower speed and fewer obstacles, the decline rate of the three performances—navigation speed, efficiency, and success rate of our algorithm is much lower than that of the other three methods, showing strong robustness.

#### 4.3.2. Assessment of obstacle avoidance ability

In terms of obstacle avoidance ability, when the dynamic obstacle speed is 0.1 m/s, the three methods have advantages over each other under the different number of dynamic obstacles. When the *v*_*obs*_ increases to 0.2 m/s, the collision times of our approach are all the lowest under different amounts of blocks. Although our method is only 2 times less than the average collision number of the TEB algorithm when there are only 5 dynamic obstacles, the gap keeps widening with the increase of obstacles. When the number of obstacles is increased to 20, the average collision number of our method is 10 times less than that of the second-ranked TEB algorithm. When the dynamic obstacle speed reaches the fastest 0.3 m/s, the gap between the other methods and the method in this research is further widened. The collision times are more than 50% more than our method. In general, our approach has the most obvious advantage in obstacle avoidance ability compared to the other three indexes—navigation speed, efficiency, and success rate. When the *v*_*obs*_ is increased to 0.2 m/s and 0.3 m/s, the obstacle avoidance ability of our algorithm is much superior to the other three algorithms.

#### 4.3.3. Overall performance

As seen from Figures 7A–C, our method presents excellent advantages when the *v*_*obs*_ is 0.3 m/s. Only the navigation efficiency is exceeded by TEB when the number of dynamic obstacles is 5. In other cases, our proposed method achieves the best performance. In addition, to more intuitively evaluate the performance of various methods under any conditions, the data in Table 1 are integrated and normalized in Figure 7D. The performance of other methods under the same conditions is divided by the corresponding performance of our algorithm to obtain the relative value and then superimposed to get the overall relative value. At the same speed, the relative values of different dynamic obstacles are averaged to obtain the normalization performance when the speed changes. When the *v*_*obs*_ is 0.1 m/s, TEB performs the best, CADRL performs almost as well as our method, and DWA performs the worst. As the speed of dynamic obstacle increases, the performance of our method begins to approach TEB. When the *v*_*obs*_ is 0.3 m/s, the performance of our method is better than the other three methods, followed by TEB and CADRL. Therefore, it can be concluded that when there are many fast-moving dynamic obstacles, our navigation framework proposed in this research can achieve high navigation efficiency, high navigation speed, high safety, and strong robustness.

#### 4.3.4. Assessment of the memory module

In this set of experiments, we evaluate our modified A3C network (visualized in Figure 4). In the same configuration environments, our method without memory module is deployed in turbobot3 and simulation experiments are carried out. Table 2 below presents experimental results.

**Table 2:** Quantitative evaluations.

	**Time[s]**	**Path[m]**	**Collisions**	**Success**	**Time[s]**	**Path[m]**	**Collisions**	**Success**	**Time[s]**	**Path[m]**	**Collisions**	**Success**
	**5 dyn. obstacles**				**10 dyn. obstacles**				**20 dyn. obstacles**			
*v*_*obs*_ = 0.1*m*/*s*												
Our method	121.19	**24.09**	**1**	100	131.69	**23.44**	7.5	**100**	**141.85**	**25.42**	14	**97**
Our method (without LSTM module)	**117.93**	26.72	2.5	100	**127.07**	25.11	**4**	97	148.29	32.21	**12**	91
*v*_*obs*_ = 0.2*m*/*s*												
Our method	**119.22**	**23.11**	3.5	**98**	**142.17**	**24.31**	**14**	**95**	**142.57**	**26.52**	26	93
Our method (without LSTM module)	127.41	27.54	**2**	92	149.38	27.89	18	92	150.33	31.76	**23.5**	**94**
*v*_*obs*_ = 0.3*m*/*s*												
Our method	**115.72**	**26.13**	**12**	**94**	**154.35**	**28.41**	**27**	**92**	**162.84**	**29.13**	44	78.5
Our method (without LSTM module)	125.49	30.04	15	89	162.29	31.94	33	85	191.41	37.52	**37**	**82**

The meaning of the bold values is the numerical value that performs best in the same dynamic environment.

**Figure 8:**
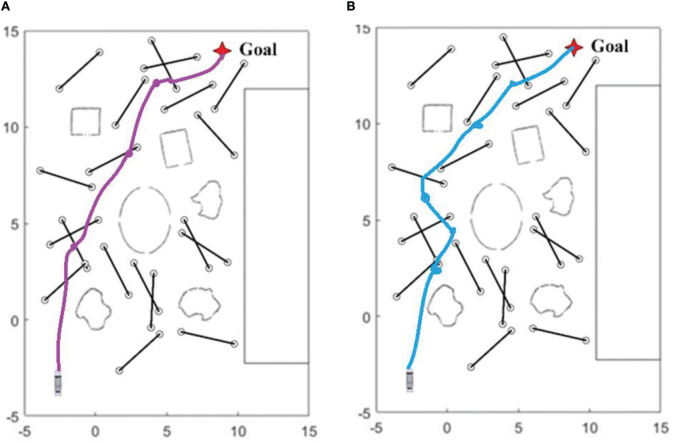
**(A)** Shows one of the trajectories of a mobile robot with a memory module deployed on a map with 20 dynamic obstacles and a speed of 0.3 m/s; and **(B)** shows one of the trajectories of a mobile robot deployed without an LSTM network on the same map.

From the table above, it can be seen that the sub-goal algorithm with memory modules slightly improves navigation success rates when there are 5 and 10 dynamic obstacles. However, we believe that what truly leverages memory modules' advantages is in terms of navigation efficiency. From the table, it can be seen that in nine different dynamic obstacle environments, algorithms with LSTM networks completed navigation to the destination in shorter time and shorter paths. In order to more intuitively demonstrate the contribution of memory modules to navigation, we recorded turbobot3's paths and selected two representative paths for comparison:

From Table 2 and Figure 8, it can be seen that compared to algorithms without memory modules deployed, memory modules can achieve better results in two aspects. Firstly, they can consider the dynamic obstacles of the global map as a whole, find the shortest possible path, and reduce unnecessary turns (even if this is to avoid obstacles); Secondly, when facing multiple moving obstacles around, the robot's ability to rotate in place is significantly reduced.

#### 4.3.5. Training results

**Figure 9:**
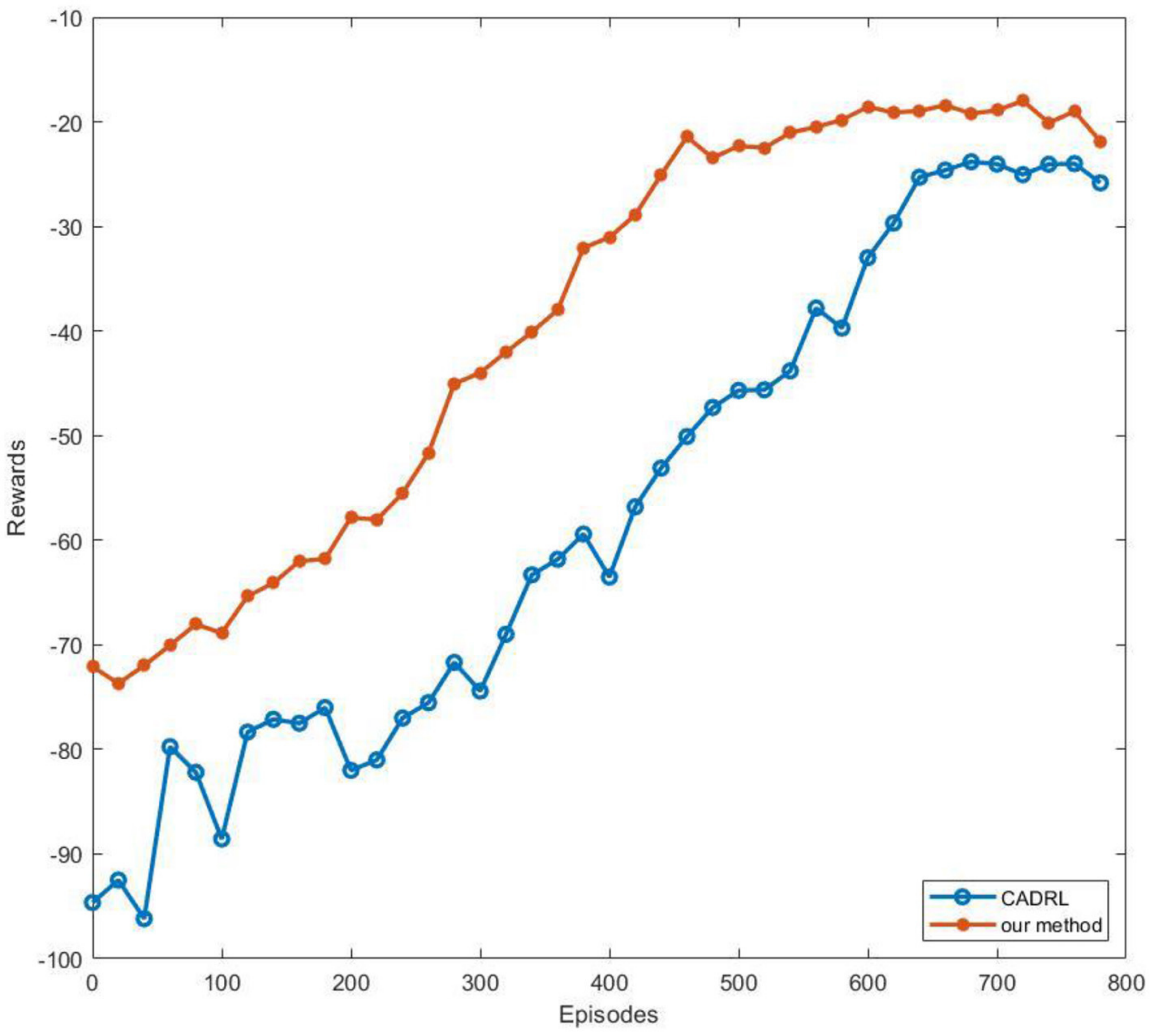
The reward convergence curve. Each network was trained with 800 episodes, and we recorded reward values as points on the line every 20 episodes. To simplify image information, we selected the maximum value of the 20 values to represent the reward.

To verify our network's advantages in rate of convergence, we trained CADRL in the same environment. At the same time, to ensure the reliability of the experiment, we kept the reward mechanism of CADRL consistent with our algorithm. We plotted the reward convergence curve of the two algorithms on a line chart, as shown in Figure 9.

From Figure 9, it can be seen that our method's reward curve begins to converge around the 470th episode, while CADRL begins to converge around the 600th episode. Compared to the end-to-end reinforcement learning navigation algorithm, our method that decomposes global navigation into global and local navigation reduces training difficulty and makes the training curve converge faster.

## 5. Conclusion

This research presents a framework for integrating DRL-based local navigation into long-distance navigation. The framework can be used for direct training and deployment of DRL-based algorithms in combination with local and traditional global planning. The landmark generator proposed in this research will generate sub-goals and significantly reduce the computational pressure of the end-to-end DRL-based navigation method. Besides integrating the traditional obstacle avoidance algorithm based on numerical optimization and reinforcement learning into the system, we use memory-enhanced A3C to construct a local trajectory planner. Then, different navigation methods were evaluated for navigation efficiency, safety, and robustness. In comparison to three traditional navigation methods and one end-to-end reinforcement learning method, our navigation framework demonstrates greater efficiency, safety, and robustness, particularly when faced with a large number of obstacles moving at speeds exceeding 0.2 m/s. In addition, the researchers of this paper will continue to explore whether the process of generating sub-goals can be included in the reinforcement learning-based decision-making process and consider more influencing factors than only finding the sub-goal closest to the final goal.

## Data availability statement

The original contributions presented in the study are included in the article/supplementary material, further inquiries can be directed to the corresponding author.

## Author contributions

XW: proposal of ideas, scheme argumentation, experimental design, code writing, and paper writing. YS: paper writing. YX: graph construction. JB: paper review. JX: resources. All authors contributed to the article and approved the submitted version.

## Footnotes

^1^The local minima in reinforcement learning refers to the behavior of the agent circling in circles not to decrease the reward value and not to conduct any navigation during the navigation process. There is no decrease or even a slight increase in reward value, but this is not what we want.

^2^Partially observable Markov decision processes (POMDP) are an ideal model for sequential decision-making in the partially known dynamic uncertain environment (Wan et al., 2018). Its core point lies in that the agent cannot see the state of its territory. Therefore, it needs to know its state by means of additional sensors or interaction with other agents so that it can objectively and accurately describe the real world and make corresponding decisions to achieve the desired purpose.

## Appendix

**Table A1:** Hyperparameters for training.

**Hyperparameter**	**Value**	**Explanation**
Continuous success threshold	10	Training considered done if the number of consecutive successes reaches this value
Discount factor	0.9	Discount factor for reward estimation (often denoted by γ)
Learning rate	0.0001	Learning rate for optimizer
Epsilon max steps	10^5^	Steps until epsilon reaches mini-mum
Epsilon end	0.003	Minimum epsilon value
Maximum gradient	0.5	Maximum value to clip the gradient
